# Pitavastatin Ameliorates Lipopolysaccharide-Induced Blood-Brain Barrier Dysfunction

**DOI:** 10.3390/biomedicines9070837

**Published:** 2021-07-18

**Authors:** Takashi Fujimoto, Yoichi Morofuji, Andrej Kovac, Michelle A. Erickson, Mária A. Deli, Masami Niwa, William A. Banks

**Affiliations:** 1 Department of Neurosurgery, Nagasaki University Graduate School of Biomedical Sciences, 1-7-1 Sakamoto, Nagasaki 852-8501, Japan; t.fujimototakashi@gmail.com; 2 Division of Gerontology and Geriatric Medicine, Department of Medicine, School of Medicine, University of Washington, Seattle, WA 98108, USA; mericks999@gmail.com (M.A.E.); wabanks1@uw.edu (W.A.B.); 3 Veterans Affairs Puget Sound Health Care System, Geriatric Research Education and Clinical Center, 1660 S. Columbian Way, Seattle, WA 98108, USA; 4 National Nagasaki Medical Center, Department of Neurosurgery, 2-1001-1 Kubara, Omura, Nagasaki 856-8562, Japan; 5 Institute of Neuroimmunology, Slovak Academy of Sciences, Dubravska cesta 9, 84510 Bratislava, Slovakia; andrej.kovac@savba.sk; 6 Biological Research Centre, Institute of Biophysics, 6726 Szeged, Hungary; deli.maria@brc.hu; 7 BBB Laboratory, PharmaCo-Cell Company, Ltd., Dai-ichi-senshu Bldg. 2nd Floor, 6-19 Chitose-machi, Nagasaki 850-8135, Japan; niwa@pharmacocell.co.jp

**Keywords:** blood-brain barrier, central nervous system diseases, cytokine, inflammation, statin, lipopolysaccharide

## Abstract

Statins have neuroprotective effects on neurological diseases, including a pleiotropic effect possibly related to blood–brain barrier (BBB) function. In this study, we investigated the effects of pitavastatin (PTV) on lipopolysaccharide (LPS)-induced BBB dysfunction in an in vitro BBB model comprising cocultured primary mouse brain endothelial cells, pericytes, and astrocytes. LPS (1 ng/mL, 24 h) increased the permeability and lowered the transendothelial electrical resistance of the BBB, and the co-administration of PTV prevented these effects. LPS increased the release of interleukin-6, granulocyte colony-stimulating factor, keratinocyte-derived chemokine, monocyte chemotactic protein-1, and regulated on activation, normal T-cell expressed and secreted from the BBB model. PTV inhibited the LPS-induced release of these cytokines. These results suggest that PTV can ameliorate LPS-induced BBB dysfunction, and these effects might be mediated through the inhibition of LPS-induced cytokine production. Clinically, therapeutic approaches using statins combined with novel strategies need to be designed. Our present finding sheds light on the pharmacological significance of statins in the treatment of central nervous system diseases.

## 1. Introduction

Statins reduce serum low-density lipoprotein (LDL) cholesterol levels by inhibiting 3-hydroxyl-3-methyl coenzyme A (HMG-CoA) reductase. Statins also have various unique pleiotropic effects in addition to cholesterol-lowering activity. Statins have been demonstrated to have beneficial effects against many central nervous system (CNS) diseases and conditions including stroke, Alzheimer’s disease, multiple sclerosis, brain trauma, and infection [1,2,3,4,5,6,7,8]. In particular, “new-generation” statins, which have a stronger cholesterol-lowering effect than conventional statins, have been developed. The new-generation statin pitavastatin (PTV) exerts potent hypolipidemic effects in both animal and human trials [9]. Reports of its pleiotropic effects on endothelial cells have also been published [10,11]. We previously reported that PTV elevated claudin-5 expression with an increase in barrier tightness in primary endothelial cell monolayer cultures [12]. However, few reports have examined the effects of statins on the blood–brain barrier (BBB) in terms of neuroinflammation.

In the present study, we investigated the effects of PTV on lipopolysaccharide (LPS)-induced BBB dysfunction in our in vitro BBB model comprising cocultured primary mouse brain endothelial cells, pericytes, and astrocytes.

## 2. Results

### 2.1. Effects of LPS on Cerebral Barrier Function

**Figure 1:**
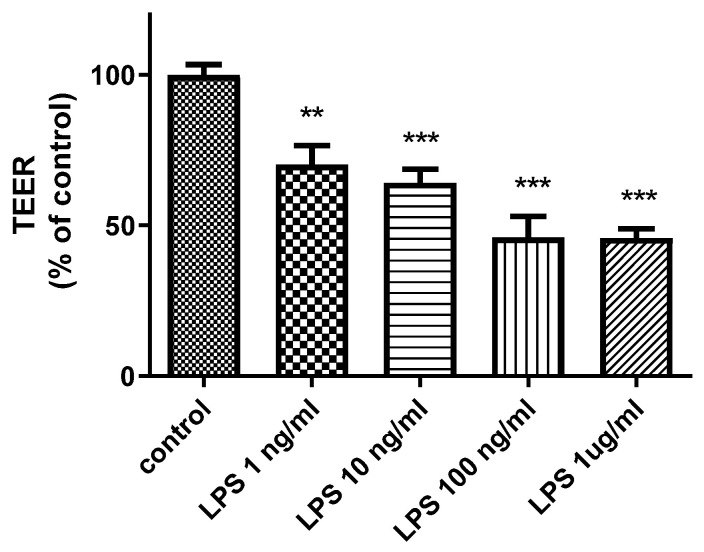
Effects of LPS on TEER in an in vitro BBB model. The figure illustrates that the addition of LPS to the luminal chamber of the in vitro BBB model for 24 h decreased TEER in a concentration-dependent manner. ** *p* < 0.01, *** *p* < 0.001 vs. control (one-way ANOVA, *p*-values were calculated from Tukey’s Multiple Comparison Test). The TEER experiment was conducted with technical replicates in triplicate experiments, *n* = 6 per group.

To analyze the potentially harmful effects of LPS on BBB integrity, we measured transendothelial electrical resistance (TEER) in an in vitro BBB model after LPS treatment. As presented in Figure 1, LPS decreased TEER in a concentration-dependent manner (*p* < 0.01).

### 2.2. Effect of PTV on BBB Function

**Figure 2:**
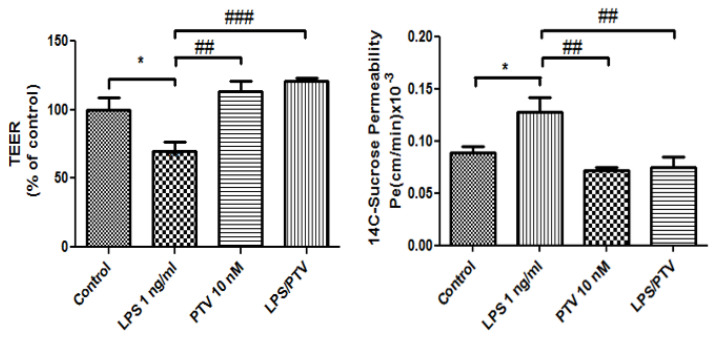
PTV restores LPS-induced changes in TEER and endothelial permeability in an in vitro BBB model. Cells were incubated with PTV in the presence or absence of LPS (added only to the luminal chamber) for 24 h. * *p* < 0.05 vs. control; ## *p* < 0.01, ### *p* < 0.001 vs. LPS (one-way ANOVA, *p*-values were calculated from Tukey’s Multiple Comparison Test). The TEER and endothelial permeability test were conducted with two technical replicates in triplicate experiments, *n* = 6 per group.

We next analyzed the protective effects of PTV on BBB function following LPS treatment in our in vitro model. As presented in Figure 2, the addition of LPS decreased TEER in the BBB model (57.8% vs. control; *p* < 0.01), and this effect was prevented by co-treatment with PTV (120.9% vs. control; *p* < 0.01). Meanwhile, treatment with PTV alone did not significantly alter TEER (113.1% vs. control; *p* = 0.29). In addition, treatment with LPS alone increased permeability in the BBB model as assessed using ^14^C-sucrose (permeability coefficient [P_e_] = 0.13 × 10^−3^ cm/min) compared to the control (P_e_ = 0.089 × 10^−3^ cm/min; *p* < 0.01), and this effect was similarly abrogated by PTV co-treatment (P_e_ = 0.075 × 10^−3^ cm/min; *p* < 0.01).

### 2.3. Secretion of Cytokines and Chemokines

We hypothesized that the effects of LPS and PTV on the BBB are largely related to cytokines. Therefore, we examined cytokine and chemokine secretion on the luminal and abluminal sides of Transwell inserts. Twenty-three cytokines and chemokines were examined comprehensively, and five of them were detected.

**Figure 3:**
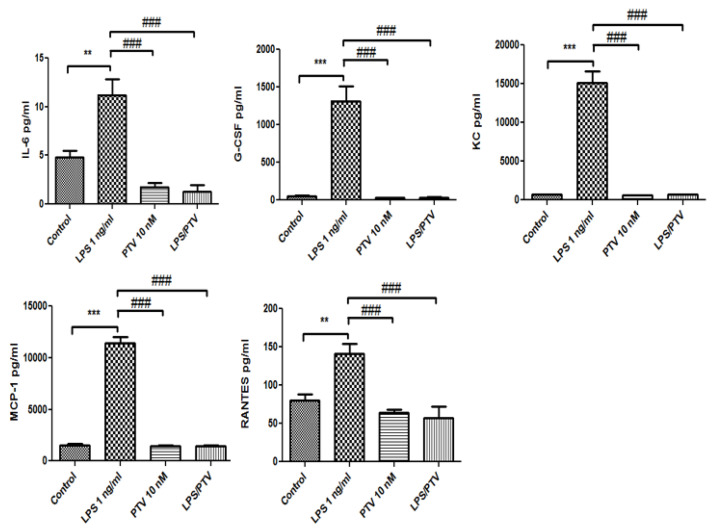
LPS increased the release of IL-6, G-CSF, KC, MCP-1, and RANTES from the in vitro BBB model into the luminal chamber. PTV inhibited the LPS-induced release of these cytokines. ** *p* < 0.01, *** *p* < 0.001 vs. control; ### *p* < 0.001 vs. LPS (two-way ANOVA).

**Figure 4:**
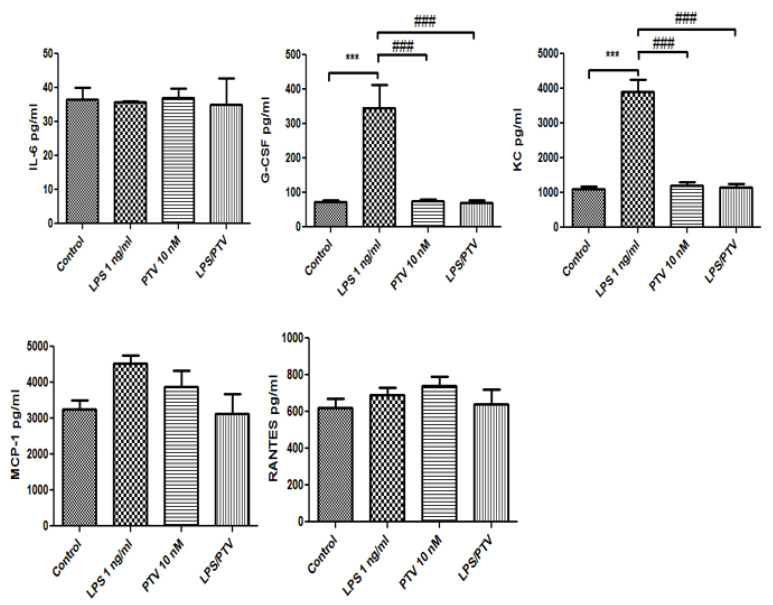
LPS increased the release of G-CSF and KC from the in vitro BBB model into the abluminal chamber, and PTV inhibited the LPS-induced release of these cytokines. The addition of LPS to the luminal chamber did not induce significant increases in the secretion of other cytokines in the abluminal chamber. *** *p* < 0.001 vs. control; ### *p* < 0.001 vs. LPS (two-way ANOVA).

Of those, LPS increased the release of interleukin (IL)-6, granulocyte colony-stimulating factor (G-CSF), keratinocyte-derived chemokine (KC), monocyte chemotactic protein-1 (MCP-1), and regulated on activation, normal T-cell expressed and secreted (RANTES) from the in vitro BBB model into the luminal chamber (all *p* < 0.01 vs. control; Figure 3). PTV inhibited the LPS-induced release of all of these cytokines (all *p* < 0.001; Figure 3). In addition, LPS increased the release of G-CSF and KC from the in vitro BBB model into the abluminal chamber (both *p* < 0.001; Figure 4), and this cytokine release was inhibited by co-treatment with PTV (both *p* < 0.001). LPS did not significantly increase the release of IL-6, MCP-1, and RANTES into the abluminal chamber.

## 3. Discussion

The anti-inflammatory effects of statins have been noted in previous studies, and therefore, we predicted that statins could counter the pro-inflammatory effects of LPS on the BBB. In the present study, we investigated the influence of LPS on BBB function and the protective effects of PTV on LPS-induced BBB dysfunction. LPS was demonstrated to damage the barrier function of the BBB model in a concentration-dependent manner. Although PTV treatment alone did not alter the barrier functions of the BBB, it counteracted the disruption caused by LPS. Additionally, PTV inhibited the LPS-induced secretion of several cytokines and chemokines, suggesting that its protective effects could be mediated through an anti-inflammatory mechanism.

LPS is a component of the cell wall of Gram-negative bacteria that by activating the innate immune system induces the production of several pro-inflammatory cytokines, subsequently damaging organs, including the brain [13]. Many cytokines are involved in LPS-induced responses, but the relationships among them are complex and incompletely understood. It is known that BBB constituent cells such as endothelial cells, astrocytes, and pericytes secrete chemokines, cytokines, and other chemicals in response to each other and that exposure to LPS increases the levels of specific cytokines [14,15]. LPS decreases the expression of BBB tight junction proteins, such as occludin and claudins, in a dose-dependent manner, thereby disturbing the BBB structure [13,16]. In addition, LPS affects BBB-related cells and increases BBB permeability [16]. LPS is also a potent activator of microglia, and the detection of peripheral inflammatory stimuli at the BBB is followed by a cascade of events leading to the activation of microglia and subsequent modulation of adjacent cells, including pericytes, astrocytes, and neurons [17]. In our study, TEER was also decreased by LPS in our BBB model in a concentration-dependent manner, suggesting the strong involvement of inflammatory effects.

Statins reduce LDL cholesterol levels by inhibiting HMG-CoA reductase, and they are useful for preventing acute coronary injury and atherothrombotic stroke. Additionally, statins exert antioxidant, anti-inflammatory, and nitric oxide (NO)-modulating activities. These NO-dependent effects are particularly involved in cerebrovascular endothelial cells, and have been reported to increase serum nitrite levels, decrease serum homocysteine, and reduce oxidative stress [18]. These drugs have beneficial effects on various CNS disorders, including Alzheimer’s disease and other neurodegenerative conditions [19,20,21]. Regarding the anti-inflammatory effects of statins, downregulation of the NF-κB pathway in endothelial cells has been observed, and this downregulation is believed to reduce the release of inflammatory cytokines and promote protective effects on the BBB [22,23,24]. In addition, statins are also known to strengthen the integrity of the BBB [12]. Our results illustrated that PTV strongly suppressed the pro-inflammatory effects of LPS on the BBB, which were related to changes in pro-inflammatory cytokine levels. Previous studies demonstrated the involvement of LPS in the release of pro-inflammatory regulators such as IL-1α, IL-1β, IL-6, IL-8, IL-9, IL-12, IL-13, IL-18, tumor necrosis factor alpha (TNF-α), G-CSF, GM-CSF, MCP-1, interferon gamma-induced protein 10, RANTES, KC, and NO from BBB-associated cells [25,26]. In mouse studies, we identified increased secretion for 15 cytokines and chemokines following LPS treatment, and among these cytokines and chemokines, IL-6, G-CSF, KC, MCP-1, and RANTES appear to be involved in the protective effects of statins on the BBB. Previous studies have suggested that these cytokines are able to directly act on brain endothelial cells, and statins were found to exert anti-inflammatory effects on endothelial cells. In addition, G-CSF and KC displayed LPS-dependent responses on the abluminal side. This indicates that the effects of statins involve both the suppression of inflammation in blood vessels and an anti-inflammatory effect in the brain parenchyma. These changes are related to the neuroprotective effects of statins after stroke and traumatic brain injury.

## 4. Materials and Methods

### 4.1. Mouse Brain Endothelial Cell Cultures

Primary mouse brain endothelial cells were prepared as described by Coisne et al. [27] with modifications. Briefly, meninges were carefully removed from the forebrains of mice, and gray matter was minced into small pieces. Preparations were pooled and ground using a Dounce homogenizer in Dulbecco’s Modified Eagle’s Medium/Nutrient Mixture F-12 Ham (DMEM/F12; Sigma-Aldrich Co., St. Louis, MO, USA) supplemented with gentamicin (50 µg/mL, Sigma-Aldrich Co., St. Louis, MO, USA). The resulting homogenate was mixed with 30% dextran (*v*/*v*, molecular weight 100,000–200,000, Sigma-Aldrich Co., St. Louis, MO, USA) in DMEM/F12 supplemented with 0.1% bovine serum albumin (BSA; Sigma-Aldrich Co., St. Louis, MO, USA). The suspension was centrifuged at 3000× *g* for 25 min at 4 °C. The pellet was suspended in DMEM/F12, and the supernatant was centrifuged again under the same conditions. After the second centrifugation, the supernatant was discarded, and the pellet was re-suspended in DMEM/F12. Then, each pellet was filtered through a 70-µm nylon mesh and digested in collagenase/dispase (2 mg/mL, Roche, Basel, Switzerland) and DNase I (10 µg/mL, Sigma-Aldrich Co., St. Louis, MO, USA) for 30 min. The digested solution was filtered through a 20-µm nylon mesh and seeded onto collagen type IV- and fibronectin-coated dishes (both from Sigma-Aldrich Co., St. Louis, MO, USA). Cultures were maintained in DMEM/F12 supplemented with 10% plasma-derived serum (PDS, Animal Technologies, Inc., Tyler, TX, USA), 1% GlutaMAX (Gibco, Thermo Fisher Scientific, Waltham, MA, USA), basic fibroblast growth factor (bFGF, Roche, Basel, Switzerland), heparin, insulin, transferrin and sodium selenite supplement, and puromycin (4 µg/mL, Sigma-Aldrich Co., St. Louis, MO, USA). Twenty-four hours after plating, red blood cells, cell debris, and nonadherent cells were removed by washing with medium. On the third day, puromycin was removed from medium. When the cultures reached 80% confluency (5th day in vitro), the purified endothelial cells were passaged by brief treatment with 0.25% trypsin-EDTA (Invitrogen, Thermo Fisher Scientific, Waltham, MA, USA) and used to construct in vitro BBB models. 

### 4.2. Mouse Brain Pericyte Culture

Primary mouse brain pericytes were prepared as described by Nakagawa et al. [28]. Briefly, the cultures of mouse cerebral pericytes were obtained via a prolonged, 2-week culture of isolated brain microvessel fragments containing pericytes and endothelial cells. Pericyte survival and proliferation were favored using selective culture conditions including uncoated dishes and DMEM/F12 supplemented with 20% fetal calf serum (Sigma-Aldrich Co., St. Louis, MO, USA), 1% GlutaMAX (Gibco, Thermo Fisher Scientific, Waltham, MA, USA), and gentamicin (Sigma-Aldrich Co., St. Louis, MO, USA). The culture medium was changed twice weekly.

### 4.3. Mouse Glial Cell Cultures

Mouse mixed glial cell cultures were prepared as described by McCarthy and de Vellis [29]. The cerebral cortices of newborn mice (0–1 day-old) were dissected, stripped off their meninges, and mechanically dissociated via repeated pipetting followed by passage through a nylon mesh. Cells were plated onto plastic dishes precoated with poly-l-lysine (10 µg/mL; Sigma-Aldrich Co., St. Louis, MO, USA) and cultivated in DMEM containing 10% fetal calf serum and 1% GlutaMAX at 37 °C and 5% CO_2_ in a water-saturated atmosphere. The medium was changed twice weekly. The cultures attained confluence after 8–10 days in vitro, and they were used between 14 and 20 days in vitro.

### 4.4. In Vitro BBB Model Generation and Treatment

Primary mouse brain pericytes (15,000 cells/cm^2^) were seeded onto the bottom of the collagen-coated polyester membrane (0.33 cm^2^, 0.4 µm pore size) of Transwell^®^ inserts (24-well type, Sigma-Aldrich Co., St. Louis, MO, USA). The pericytes were allowed to adhere firmly overnight. Endothelial cells (150,000 cells/cm^2^) were seeded the next day on the inside of the inserts. The inserts were then placed in the wells of the 24-well plate cultured with astrocytes. In vitro BBB models were maintained in DMEM/F12 supplemented with 10% PDS, 1% GlutaMAX, bFGF, heparin, insulin, transferrin, and sodium selenite supplemented with hydrocortisone (500 nM; Sigma-Aldrich Co., St. Louis, MO, USA) at 37 °C in a humidified atmosphere of 5% CO_2_ and 95% air. Experiments were performed 3 or 4 days after endothelial cells were seeded. For stimulation, experiment cells were washed with serum-free medium and exposed to culture medium with or without LPS from *Salmonella enterica* serovar Typhimurium (L6511; Sigma-Aldrich Co., St. Louis, MO, USA) for 24 h. PTV was added into the luminal chamber (upper compartment) of the BBB model constructed with Transwell. Based on our previous report, we used a PTV concentration of 10 nM. 

### 4.5. TEER and Sucrose Permeability

TEER (in Ω × cm^2^) was measured using an EVOM resistance meter (World Precision Instruments, Sarasota, FL, USA). The TEER of cell-free inserts was subtracted from the measured values. For the transport experiments, the medium was removed, and inserts were washed with Krebs–Ringer phosphate HEPES (KRPH) buffer containing 1% BSA (141 mM NaCl, 4.0 mM KCl, 2.8 mM CaCl_2_, 1.0 mM MgSO_4_, 1.0 mM NaH_2_PO_4_, 10 mM HEPES, 10 mM D-glucose and 1% BSA, pH 7.4). KRPH buffer containing 1% BSA was added to the abluminal chamber of the Transwell^®^ insert. To initiate the transport experiments, ^14^C-sucrose (1.5 × 10^6^ cpm/mL; PerkinElmer, Waltham, MA, USA) was loaded on the luminal chamber. Samples were removed from the abluminal chamber after 10, 20, 30, and 45 min and immediately replaced with an equal volume of fresh 1% BSA/KRPH buffer. The radioactivity of ^14^C-sucrose was measured using a liquid scintillation counter (Packard Tri-Carb 1900, PerkinElmer, Waltham, MA, USA). The P_e_ and clearance of ^125^I-albumin and ^14^C-sucrose were calculated as described by Dehouck et al. [30].

Clearance was expressed as the amount of the radioactive tracer diffusing from the luminal chamber to the abluminal chamber and calculated using the initial amount of radioactivity in the loading chamber and the measured amount of radioactivity in the collected samples. The formula for clearance is as follows:

Clearance (μL) = [C]_C_ × V_C_/[C]_L_,

where [C]_L_ is the initial amount of radioactivity per microliter of the solution loaded into the insert (in counts per minute per microliter), [C]_C_ is the radioactivity per microliter in the collected sample (in counts per minute per microliter), and V_C_ is the volume of collecting chamber (in microliters). The clearance volume increased linearly with time.

The volume cleared was plotted versus time, and the slope was estimated via linear regression analysis. The slope of clearance curves for the BMEC monolayer plus Transwell membrane was denoted by PS_app_, where PS is the permeability × surface area product (in microliters per minute). The slope of the clearance curve with a Transwell membrane without BMECs was denoted by PS_membrane_. The PS value for the BMEC monolayer (PS_e_) was calculated as follows:

1/PS_app_ = 1/PS_membrane_ + 1/PS_e_.

PS_e_ was divided by the surface area of the Transwell inserts to generate the endothelial P_e_ (in microliters per minute per square centimeter).

### 4.6. Enzyme-Linked Immunosorbent Assay (ELISA) of Cytokines

The concentrations of mouse cytokines and chemokines secreted into the culture medium were measured using a commercial magnetic bead-based Multiplex ELISA kit (Bioplex, Bio-Rad, Hercules, CA, USA) according to the manufacturer’s protocol. Of the 23 cytokines in the mouse kit, we detected 15 different cytokines: IL-1α, IL-1β, IL-6, IL-12 (p40), IL-12 (p70), IL-13, G-CSF, GM-CSF, eotaxin, KC, MCP-1, macrophage inflammatory protein (MIP)-1α, MIP-1β, chemokine (C–C motif) ligand 5 (RANTES), and TNF-α.

### 4.7. Data Analysis

All experiments were performed in triplicate and repeated once (*n* = 6). Values are presented as the mean ± SEM. Statistical analysis was performed by two-way ANOVA using GraphPad Prism (GraphPad, San Diego, CA, USA). The Newman–Keuls multiple comparison test was used for post hoc comparisons. *p* < 0.05 denoted statistical significance.

## 5. Conclusions

Our results suggest that PTV can ameliorate LPS-induced BBB dysfunction, and this effect might be mediated through the inhibition of LPS-induced cytokine and chemokine production. Clinically, therapeutic approaches using statins together with novel strategies need to be designed. Our present findings shed light on the pharmacological significance of statins in the treatment of CNS diseases.

## Data Availability

The data that support the findings of this study are available from the corresponding author upon reasonable request.
